# External validation of the joint-specific bone involvement, antimicrobial options, coverage of the soft tissues, and host status (JS-BACH) classification for predicting the outcome in periprosthetic joint infections following total hip and knee arthroplasties: a promising tool for clinical practice

**DOI:** 10.5194/jbji-10-501-2025

**Published:** 2025-11-28

**Authors:** Benjamin Schlossmacher, Vincent Lallinger, Dirk Müller, Rüdiger von Eisenhart-Rothe, Igor Lazic

**Affiliations:** 1 Department of Orthopaedics and Sports Orthopaedics, TUM Universitaetsklinikum, Klinikum rechts der Isar, Ismaninger Str. 22, 81675 Munich, Germany

## Abstract

**Purpose**: Periprosthetic joint infection (PJI) represents a major complication of total joint arthroplasty (TJA). The joint-specific bone involvement, antimicrobial options, coverage of the soft tissues, and host status (JS-BACH) classification of 2021 aims to categorize PJI severity and predict PJI recurrence and quality of life following surgical PJI treatment. Until now, only one external validation has confirmed its predictive value for treatment failure. This study aimed to further validate the classification in an external cohort and to compare outcomes between different pathogen groups. **Methods**: We applied the JS-BACH classification to a cohort of 249 consecutive gram-positive (staphylococci) and gram-negative PJIs in hip and knee joints treated at our institution between 2010 and 2022 (*Staphylococcus aureus* n=62 ; coagulase-negative staphylococci n=115 ; gram-negative organisms n=72 ). According to the JS-BACH classification, we divided cases into uncomplicated ( n=35 ), complex ( n=155 ), and limited options ( n=59 ). The median (interquartile range, IQR) follow-up was 25.0 (3–59) and at least 12 months. Outcomes were assessed based on the 2013 Delphi consensus on PJI outcome. PJI was defined following the EBJIS classification. **Results**: A higher JS-BACH category correlated significantly with a lower infection-free survival. Using uncomplicated cases as baseline, the hazards ratio (HR) was 3.2 (95 %-CI 1.3–7.9) for complex and 6.6 (95 %-CI 2.6–16.7) for limited options cases. Similarly, higher JS-BACH categories were associated with lower revision-free survival for recurrent PJI, again with uncomplicated cases as baseline: complex HR 2.2 (95 %-CI 0.9–5.5); limited options HR 4.1 (95 %-CI 1.6–10.8). The mean infection-free survival was 85.7 %, 58.7 %, and 33.9 % for uncomplicated, complex, and limited options cases ( p<0.001 ). **Conclusion**: The novel JS-BACH classification provides reliable predictions of treatment outcome for the proposed subgroups. It provides a structured and simple-to-use option for classifying PJI in daily clinical practice and for scientific purposes.

## 1 Introduction

Despite the generally positive outcomes of total joint arthroplasty (TJA), the procedure's increasing frequency in recent years has been accompanied by a significant rate of complications, resulting in implant revision rates as high as 20 % (Singh, 2011; Kurtz et al., 2005; Corbett et al., 2010).

One of the most severe complications following TJA is a periprosthetic joint infection (PJI).

Parts of the current research on PJI focus on a more individualized and targeted approach for treatment optimization. Several risk assessment tools have been proposed in the literature, e.g. the so-called KLIC-score and the CRIME-80-score, both predicting treatment outcome and identifying patients at risk for failure following debridement, antibiotics, and implant retention (DAIR) (Tornero et al., 2015; Wouthuyzen-Bakker et al., 2019). The KLIC-score is tailored towards early acute PJI, and the CRIME-80-score refers to late acute (haematogenous) PJI.

In 2021, Hotchen et al. proposed the so-called joint-specific bone involvement, antimicrobial options, coverage of the soft tissues, and host status (JS-BACH) classification (Hotchen et al., 2021). It represents a classification system on the severity of PJI to predict (1) the recurrence of PJI and (2) the quality of life following surgical PJI treatment. It derived from the original BACH classification on osteomyelitis of long bones (Hotchen et al., 2019) and was adapted adding the JS for joint-specific, while B for bone involvement was retained for the integrity of the acronym but does not represent a category for PJI cases.

The work group found a significant correlation between a higher JS-BACH category and both a higher rate of PJI recurrence and a lower quality of life (EQ-5D).

Until now, only one external validation on a cohort of 650 patients from Australia and New Zealand has confirmed the initially reported correlation between higher categories and higher recurrence rates (Kristensen et al., 2024).

The JS-BACH classification seems to offer a significant improvement for identifying patients at risk of PJI treatment failure in daily clinical practice and optimize targeted treatment, but further confirmation is necessary.

Therefore, the aim of this study is to (1) perform an external validation of the proposed JS-BACH classification on the prediction of PJI recurrence and (2) compare the reliability for different pathogen subgroups of causative organisms.

## 2 Material and methods

We conducted an external validation of the novel JS-BACH classification on a retrospective cohort of 249 consecutive cases of gram-positive (staphylococci) and gram-negative PJI in total hip and knee arthroplasty (THA and TKA) treated at an academic tertiary referral centre between 2010 and 2022.

PJI was defined following the EBJIS criteria (McNally et al., 2021). Following the classification, only confirmed infections (e.g. sinus tract, leukocyte count > 3.0 G L^−1^, two positive intraoperative samples with the same organism) were included. All data were drawn retrospectively from a comprehensive database for all PJIs treated at our institution. Inclusion criteria were a minimum follow-up of 1 year and fully available data sets.

### 2.1 JS-BACH classification

We evaluated the severity of all included cases following the initially presented JS-BACH classification (Table A1). The evaluation was performed by one orthopaedic surgeon with extensive experience in the field of PJI. All data were drawn from an extensive, retrospective database on all PJIs treated at our institution.

The classification comprises four subgroups that are divided into categories of increasing severity (uncomplicated, complex, limited options):

1. “ J ” relates to the involved implant size (primary, revision, or tumour implant), a possible loosening, and bone loss. For *J1–3*, we adhered to the categories specified by Hotchen et al. involving the implant design and possible loosening or bone loss.
2. “ A ” relates to antimicrobial options and potential resistances of the causative pathogens. For *A1–3*, we also adhered to the original categories. Isolates susceptible to more than 80 % of all tested antimicrobial agents were accounted as uncomplicated cases. Isolates that were resistant against more than 20 % of all antimicrobial agents or showed no susceptibility towards biofilm-active substances were accounted as complex. Lastly, isolates that were susceptible to no or only one antimicrobial agent represented the limited options group.
3. “ C ” relates to the soft tissue coverage and possible need for plastic surgery. *C1–2* represented uncomplicated or complex cases where plastic surgery for closure of the soft tissues was either necessary or not. There was no limited options group for closure in the original JS-BACH classification.
4. “ H ” relates to the host status and possible severe co-morbidities of the patient.

The highest category defines the overall JS-BACH category; e.g. a megaprosthesis results in a limited options case irrespective of microbial, soft-tissue, or patient factors.

### 2.2 Treatment algorithms

Prior to revision of the implant, detailed case assessment took place and treatment options were discussed with both an interdisciplinary team and the patient. Team members were the responsible orthopaedic surgeon, a microbiologist, and a pharmacist.

Surgical treatment included DAIR or single-, two-, or multi-stage revision depending on the general health status of the patient and the type of infection. Two-stage revision involved a 6-week interval with an antibiotic-loaded polymethylmethacrylate spacer (gentamicin, vancomycin, and/or meropenem) and systemic antibiotics. DAIR procedures explicitly included the replacement of all mobile components. Antibiotic therapy was continued for a duration of 12 weeks.

Intraoperatively, standardized culture biopsies, sonication of the implant, and one histopathological biopsy were acquired.

### 2.3 Outcome measures

Treatment was considered successful according to the Delphi-based International Multidisciplinary Consensus consisting of three features: (1) healed wound without fistula, drainage, or pain, indicating infection eradication and no infection recurrence caused by the same organism strain; (2) no subsequent surgical intervention for infection after re-implantation surgery (PJI-revision-free survival); and (3) no occurrence of PJI-related mortality (Diaz-Ledezma et al., 2013).

### 2.4 Ethics and statistical analysis

The study was approved by the local institution's Ethics Committee (reference no. 714/20 S) and was conducted in accordance with the Helsinki Declaration.

Patients' written consent was obtained in advance. Follow-up took place through out-patient clinic visits.

Normally distributed variables are given as the mean and standard deviation (SD); non-normally distributed variables are given as the median and interquartile range (IQR). The Shapiro–Wilk test was used to assess whether the variables followed a normal distribution.

For non-normally distributed variables, a Mann–Whitney U test and a t test for normally distributed variables were performed for all continuous variables. Pearson's $\chi^{2}$ tests were performed for comparison of categorical variables. Values of α<0.05 were considered to indicate statistical significance. Survival analysis was done using Kaplan–Meier survival statistics. A log-rank test and Cox regression were used for survival comparisons. Hazards ratios (HRs) are given with 95 % confidence intervals in brackets.

Statistical analysis and generation of all figures was carried out using IBM SPSS Statistics for Windows, version 27.0 (IBM Corporation, Armonk, NY, USA).

## 3 Results

### 3.1 Demographics

A total of 249 cases were included in the following study population. Median (IQR) follow-up was 25.0 (3–52) months. Cases without treatment failure were followed for at least 12 months. There were 35 uncomplicated (14.1 %), 155 complex (62.2 %), and 59 limited options cases (23.7 %). Detailed patient demographics are demonstrated in Table 1.

**Table 1:** Patients' demographics and data on periprosthetic infections divided by categories of the joint-specific bone involvement, antimicrobial options, coverage of the soft tissues, and host status classification (JS-BACH). ^*^ DAIR = debridement, antibiotics, and implant retention; IQR = interquartile range; SD = standard deviation. Bold values denote p<0.05 .

	All cases	Uncomplicated	Complex	Limited	p value
	( n=249 )	( n=35 )	( n=155 )	options	
				( n=59 )	
Age in years (median, IQR)	70.0 (61–77)	68.0 (58–73)	71.0 (63–77)	70.0 (58–76)	0.128
Sex ( n , %)					0.619
Male	112 (45.0)	17 (48.6)	66 (42.6)	29 (49.2)	
Female	137 (55.0)	18 (51.4)	89 (57.4)	30 (50.8)	
Location ( n , %)					0.421
Knee	81 (32.5)	9 (25.7)	55 (35.5)	17 (28.8)	
Hip	168 (67.5)	26 (74.3)	100 (64.5)	42 (71.2)	
Follow-up in months (median, IQR)	25.0 (3–59)	31.0 (24–47)	30.0 (5–60)	6.0 (1–41)	**0.001**
ASA classification ( n , %)					
ASA I	11 (4.4)	5 (14.3)	6 (3.9)	–	**0.036**
ASA II	123 (49.4)	23 (65.7)	68 (43.9)	32 (54.2)	0.383
ASA III	115 (46.2)	7 (20.0)	81 (52.2)	27 (45.8)	0.068
BMI in kg m^−2^ (median, IQR)	27.1 (24.2–32.0)	27.9 (24.4–32.1)	27.1 (24.6–31.6)	27.8 (24.1–33.3)	0.973
Type of infection (Tsukayama et al., 1996) ( n ; %)					
Early acute	57 (22.9)	12 (34.3)	34 (21.9)	11 (18.6)	0.196
Acute haematogenous	26 (10.4)	4 (11.4)	14 (9.0)	8 (13.6)	0.613
Late chronic	159 (63.9)	18 (51.4)	103 (66.5)	38 (64.4)	0.246
Positive intraoperative cultures	7 (2.8)	1 (2.9)	4 (2.6)	2 (3.4)	0.950
Type of definitive therapy ( n , %)					
DAIR^*^	85 (34.1)	14 (40.0)	54 (34.8)	17 (28.8)	0.519
Single-stage revision	21 (8.4)	1 (2.9)	9 (5.8)	11 (18.6)	**0.005**
Two-stage revision	98 (39.4)	16 (45.7)	63 (40.6)	19 (32.2)	0.374
Multi-stage revision	45 (18.1)	4 (11.4)	29 (18.7)	12 (20.3)	0.524
Implant types ( n , %)					
Primary arthroplasty	91 (36.5)	35 (100.0)	48 (31.0)	8 (13.6)	< **0.001**
Revision arthroplasty	122 (49.0)	–	107 (69.0)	15 (25.4)	< **0.001**
Megaprostheses	36 (14.5)	–	–	36 (61.0)	< **0.001**
Microbiology					
*Staphylococcus aureus*	62 (24.9)	10 (28.6)	36 (23.2)	16 (27.1)	0.726
Coagulase-negative staphylococci	115 (46.2)	11 (31.4)	85 (54.8)	19 (32.2)	**0.002**
Gram-negative organisms	72 (28.9)	14 (40.0)	34 (21.9)	24 (40.7)	**0.008**
Polymicrobial infections ( n , %)	67 (26.9)	10 (28.6)	37 (23.9)	20 (33.9)	0.326
Prior surgeries (mean, SD)	2 (1–4)	2 (1–2)	2 (1–4)	2 (1–5)	0.874
Prior PJI in history ( n , %)	84 (33.7)	5 (14.3)	52 (33.5)	27 (45.8)	**0.008**

### 3.2 Microbiology

Coagulase-negative staphylococci were the most frequent pathogen (115/249; 46.2 %), followed by gram-negative organisms (72/249; 28.9 %) and *Staphylococcus aureus* (62/249; 24.9 %). There were 67 polymicrobial infections (26.9 %).

### 3.3 Treatment modalities

Surgical procedures included DAIR (85/249; 34.1 %) and two-stage (98/249; 39.4 %), multi-stage (45/249; 18.1 %) and single-stage revision (21/249; 8.4 %).

### 3.4 Outcome

The overall infection- and PJI-revision-free survival was 56.6 % (141/249) and 70.7 % (176/249), respectively.

Two-stage revision provided a significantly higher infection-free survival of 71.4 % (70/249) in comparison to DAIR (48.2 %; 41/85), single-stage revision (47.6 %; 10/21), and multi-stage revision (44.4 %; 20/45) ( p=0.003 ).

PJIs caused by coagulase-negative staphylococci and *Staphylococcus aureus* were both associated with an improved infection-free survival when compared to gram-negative PJI. HR was 0.4 (0.2–0.8) and 0.4 (0.3–0.7), respectively.

In total, 18 amputations (7.2 %), 17 resection arthroplasties (6.8 %), and 2 arthrodeses (0.8 %) had to be performed for definitive infection control but all only after prior staged revisions.

The risk for amputation following PJI treatment was significantly higher for complex (9/155; 5.8 %) and limited options cases (9/59; 15.3 %) in comparison to the uncomplicated group where no amputation had to be performed. The odds ratio (OR) was 0.8 (0.7–0.9) and 0.6 (0.5–0.7), respectively.

### 3.5 JS-BACH validation

**Figure 1:**
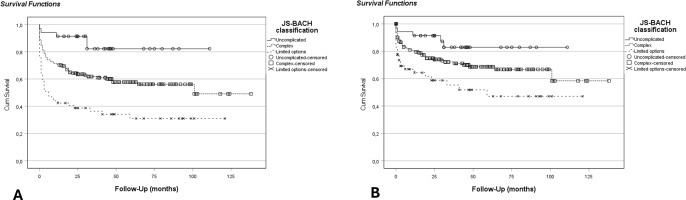
Kaplan–Meier analysis for the comparison of JS-BACH categories: **(a)** infection-free survival ( p<0.001 ), where HR was 3.2 (CI 1.3–7.9) for complex and 6.6 (CI 2.6–16.7) for limited options cases compared to uncomplicated cases; **(b)** PJI-revision-free survival ( p=0.003 ), where HR was 2.2 (CI 0.9–5.5) for complex and 4.1 (CI 1.6–10.8) for limited options cases compared to uncomplicated cases.

A higher overall JS-BACH category significantly correlated with a lower infection-free survival in the univariate Cox regression model. HR was 3.2 (1.3–7.9) for complex and 6.6 (2.6–16.7) for limited options cases. Additionally, the limited options group showed a significantly lower PJI-revision-free survival than uncomplicated cases with an HR of 4.1 (1.6–10.8). PJI-revision-free survival in complex cases did not significantly differ from uncomplicated cases (HR 2.2; 0.9–5.5) (Fig. 1).

**Figure 2:**
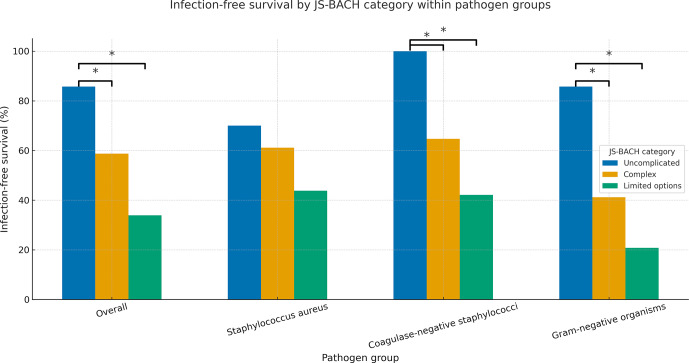
Treatment success comparison between JS-BACH categories and separate subgroup analysis of *Staphylococcus aureus*, coagulase-negative staphylococci, and gram-negative pathogens. ^*^ indicates significant differences ( p<0.05 ).

For microbial subgroup analysis, a higher JS-BACH category correlated significantly with a lower infection-free survival within gram-negative and coagulase-negative PJI (Table 2; Fig. 2).

**Table 2:** Treatment success comparison between JS-BACH categories and separate subgroup analysis of *Staphylococcus aureus* (STAU), coagulase-negative staphylococci (CNS) and gram-negative pathogens (GN). 1 = uncomplicated; 2 = complex; 3 = limited options; HR = hazards ratio; Ref. = reference/baseline; n/a = not applicable: no HR is given, as Cox regression was not applicable, with no failures appearing in the CNS-1 group. Bold values denote p<0.05 .

Causative	JS-BACH	Overall	Univariate HR for	p	Overall PJI-	Univariate HR for	p	
pathogen	category	infection-free	infection-free	value	revision-free	revision-free	value	
		survival (%)	survival (95 %-CI)		survival (%)	survival (95 %-CI)		
Overall		141/249 (56.6)	–	–	176/249 (70.7)	–	–	
	1	30/35 (85.7)	Ref.	Ref.	30/35 (85.7)	Ref.	Ref.	
	2	91/155 (58.7)	3.2 (1.3–7.9)	**0.003**	111/155 (71.6)	2.2 (0.9–5.5)	0.085	
	3	20/59 (33.9)	6.6 (2.6–16.7)	< **0.001**	35/59 (59.3)	4.1 (1.6–10.8)	**0.007**	
STAU		36/62 (58.1)	–	–	48/62 (77.4)	–	–	
	1	7/10 (70.0)	Ref.	Ref.	8/10 (80.0)	Ref.	Ref.	
	2	22/36 (61.1)	1.4 (0.4–4.9)	0.606	26/36 (72.2)	1.5 (0.3–7.0)	0.620	
	3	7/16 (43.8)	2.3 (0.6–8.4)	0.191	14/16 (87.5)	0.8 (0.1–5.4)	0.606	
CNS		74/115 (64.3)	–	–	88/115 (76.5)	–	–	
	1	11/11 (100.0)	Ref.	Ref.	11/11 (100.0)	Ref.	Ref.	
	2	55/85 (64.7)	n/a	**0.041**	65/85 (76.5)	n/a	0.314	
	3	8/19 (42.1)	n/a	**0.003**	12/19 (63.2)	n/a	0.067	
GN		31/72 (43.1)	–	–	41/72 (56.9)	–	–	
	1	12/14 (85.7)	Ref.	Ref.	12/14 (85.7)	Ref.	Ref.	
	2	14/34 (41.2)	5.2 (1.2–22.3)	**0.005**	20/34 (58.8)	3.5 (0.8–15.7)	0.072	
	3	5/24 (20.8)	8.3 (1.9–35.6)	< **0.001**	9/24 (37.5)	6.2 (1.4–27.5)	**0.004**	

Within the coagulase-negative subgroup, a Kaplan–Meier analysis was performed, as a Cox regression was not applicable, as there were no failures within the uncomplicated category (baseline – BL: uncomplicated; complex p=0.041 ; limited options p=0.003 ).

For *Staphylococcus aureus*, a non-significant correlation between a higher JS-BACH category and worse outcomes was found (Table 2; Fig. 2).

## 4 Discussion

### 4.1 Validation and predictive value

The most important finding of this study is that the JS-BACH classification system is a valid predictor of infection-free survival in patients with a periprosthetic joint infection, confirming its prognostic value across a second, independent cohort. Our results align with and further support the findings of Hotchen et al. (2021) and the prior external validation by Kristensen et al. (2024), demonstrating a clear correlation between increasing JS-BACH severity categories and worse clinical outcomes. Specifically, patients classified as “complex”, or “limited options” had a significantly higher risk for infection recurrence compared to those in the “uncomplicated” category. This pattern was consistent both in the overall cohort and within specific pathogen subgroups, especially for gram-negative organisms and coagulase-negative staphylococci (CNS). A similar but non-significant tendency was found for PJIs caused by *Staphylococcus aureus*, probably due to the smaller sample size.

The definition of treatment success and infection recurrence varied in the other two studies on the JS-BACH classification. While the Australian PIANO cohort defined treatment failure as (1) death, (2) clinical or microbiological signs of infection, (3) removal of destination prosthesis, or (4) ongoing antibiotic use within 2 years after PJI treatment, the JS-BACH group regarded treatment failure as any PJI recurrence following the EBJIS definition of 2021 (McNally et al., 2021). Previous studies have shown a significant difference in treatment success rates when applying different definitions, e.g. ranging between 54 %–89 % for the same cohort (Tan et al., 2018; Debbi et al., 2024). As a consequence, we reviewed a possible correlation between a higher JS-BACH category and the risk of revision for recurrent PJI. While complex cases did not show a statistically significant correlation in comparison to uncomplicated ones, the limited options group did have a significant 4-times elevated risk of surgical revision following PJI treatment. We therefore emphasize that the JS-BACH classification can also be validated in terms of revision-free survival in this cohort.

### 4.2 Comparison to other PJI scoring systems

As initially mentioned, several different scoring systems for predicting PJI outcome have been proposed. While the KLIC- and Crime-80-score are reserved for predicting treatment success following DAIR and only the KLIC-score has been validated externally with widely ranging results (Tornero et al., 2015; Wouthuyzen-Bakker et al., 2019), there are two other systems trying to categorize the severity of PJI in general: the PJI-TNM system of 2020 (Alt et al., 2020) and the McPherson classification from 2002 (McPherson et al., 2002). While the PJI-TNM system has not been extensively utilized yet, the McPherson classification is frequently reported in PJI study cohorts (Coughlan and Taylor, 2020). However, its clinical utility remains limited due to several shortcomings. Notably, there is a lack of robust external validation studies supporting its reliability (Coughlan and Taylor, 2020). Additionally, it does not account for key microbiological variables, such as the causative organisms and their antimicrobial resistance profiles. While the inclusion of distinct systemic and local wound factors aims to stratify patient risk, these parameters are narrowly defined and may not capture the full spectrum of relevant clinical variables. Hence, its clinical applicability remains suboptimal in daily practice.

### 4.3 Clinical practice

From a clinical perspective, our and previous findings emphasize the relevance of stratifying PJI patients not just by clinical features alone but also by microbiological and host factors captured in the JS-BACH system and put them into simple-to-follow categories. Given its simplicity and applicability across a broad cohort of PJI cases, the JS-BACH classification may be integrated into multidisciplinary decision-making algorithms to better tailor treatment strategies. For instance, patients with “limited options” scores may benefit from earlier consideration of aggressive or non-standard treatment approaches and referral to specialized PJI centres as proposed by Hotchen et al. This study further demonstrates that the JS-BACH classification exhibits germ-specific relevance, with particularly notable associations observed in PJI caused by gram-negative bacteria and CNS. These results highlight the influential role of the microbiological profile, which remains insufficiently addressed within the current structure of the “antimicrobial options” category. Future studies should further explore the potential for microbiologically driven stratification within the JS-BACH framework to enhance its predictive accuracy and clinical relevance.

### 4.4 Comparison between JS-BACH, PIANO, and our cohort

Our results showed a success rate of 56.6 %, while the original JS-BACH study and the external validation study by Kristensen et al. (2024) reported success rates of 66.4 % and 62.0 %, respectively.

The most likely explanation is the relative overrepresentation of complex and limited options cases in our cohort. They made up 85.7 % of all cases in comparison to 76.3 % in the original JS-BACH cohort and 59 % in the external validation PIANO cohort.

It is important to note that both prior studies were conducted using multi-centre cohorts – specifically, data from 2 hospitals in the UK and 27 hospitals across Australia and New Zealand. In contrast, the present study draws from a single tertiary academic referral centre yet still comprises a comparatively high case volume. Notably, this cohort includes the highest absolute and relative proportion of “limited options” cases reported to date. Additionally, the extended inclusion period enhances the robustness of the data and minimizes the risk of selection and performance bias. Therefore, the external validation of the JS-BACH classification in this study is likely to reflect the clinical realities encountered in high-volume referral centres.

## 5 Limitations

Several limitations of this study should be acknowledged. Firstly, the study's retrospective design inherently carries limitations, including potential biases and data collection challenges.

Secondly, due to lack of collected outcome measures for the patients' quality of life, we were not able to perform a validation on the predictive value of the JS-BACH classification on this matter. While the results published by Hotchen et al. (2021) seem promising, further external validation on this matter is necessary.

Due to the lack of a multi-observer assessment and inter-observer analysis, there is no verification for possible deviations in case assessment between different observers. For further evaluations, an analysis of the classification for inter-observer reliability is of key importance.

Lastly, the relatively short follow-up duration restricts our ability to draw conclusions about long-term clinical outcomes. However, a relevant number of infections have already recurred within a short interval, and we therefore decided not to exclude further cases.

## 6 Conclusion

This study confirms the prognostic value of the JS-BACH classification in predicting treatment outcomes for periprosthetic joint infections. Higher JS-BACH categories were associated with significantly lower infection- and revision-free survival. Our findings further underscore the germ-specific validity of the JS-BACH classification, particularly in gram-negative and CNS PJI. The classification provides a practical and reliable tool for risk stratification and treatment planning in daily clinical practice. Further research is warranted to evaluate the integration of microbiological profiles into the JS-BACH classification and to assess the inter-observer reliability.

## Data Availability

The data used in this work are available from the corresponding author upon request.

## Appendix A

**Table A1:** The joint-specific bone involvement, antimicrobial options, coverage of the soft tissues, and host status (JS-BACH) classification for predicting outcomes in periprosthetic joint infections. Adapted from Hotchen et al. (2021).

	JS	B	A	C	H
	Joint (prosthetic)	(Bone	Antimicrobial options	Coverage of soft	Host status
		involvement)		tissues	
Uncomplicated	J1		Ax	C1	H1
	Prosthetic joint infection with		Unknown/culture negative	Direct closure possible	Well-controlled disease or
	all of the following:			– plastic surgery	patient fit and well
	– primary-type implant in situ		A1	expertise not required	
	– minimal or no bone loss		All isolates sensitive to >80 %		
	– no evidence of loosening		of susceptibility tests and		
	– no history of periprosthetic		resistant to <3 susceptibility		
	fracture		tests		
Complex	J2		A2	C2	H2
	Prosthetic joint infection with		Any isolate either	Direct closure not	Patient with poorly controlled
	either		– sensitive to <80 % of all	possible – plastic	co-morbidity or severe
	– associated periprosthetic		susceptibility tests performed	surgery expertise	co-morbidity (evidence of end
	fracture		– resistant to ≥4 susceptibility	required	organ damage)
	– moderate bone loss		tests		or
	– prosthetic loosening		– resistant to anti-biofilm		recurrent bone infection after
	– non-primary-type implant in		antibiotics in the presence of		previous debridement
	situ		an implant		
Limited options	J3		A3		H3
	Prosthetic joint infection with		Any isolate sensitive to 0 or		Unfit for definitive surgery
	either:		1 susceptibility test		despite specialist intervention
	– custom or tumour-type				or
	implant in situ				patient declines surgery
	– custom or total bone				
	replacement required for				
	reconstruction				
	– major bone loss				

## References

[bib1.bib1] Alt V, Rupp M, Langer M, Baumann F, Trampuz A (2020). Can the oncology classification system be used for prosthetic joint infection?: The PJI-TNM system. Bone Joint Res.

[bib1.bib2] Corbett KL, Losina E, Nti AA, Prokopetz JJ, Katz JN (2010). Population-based rates of revision of primary total hip arthroplasty: a systematic review. PLoS One.

[bib1.bib3] Coughlan A, Taylor F (2020). Classifications in Brief: The McPherson Classification of Periprosthetic Infection. Clin Orthop Relat Res.

[bib1.bib4] Debbi EM, Khilnani T, Gkiatas I, Chiu Y-F, Miller AO, Henry MW, Carli AV (2024). Changing the definition of treatment success alters treatment outcomes in periprosthetic joint infection: a systematic review and meta-analysis. J Bone Joint Infect.

[bib1.bib5] Diaz-Ledezma C, Higuera CA, Parvizi J (2013). Success after treatment of periprosthetic joint infection: a Delphi-based international multidisciplinary consensus. Clin Orthop Relat Res.

[bib1.bib6] Hotchen AJ, Dudareva M, Ferguson JY, Sendi P, McNally MA (2019). The BACH classification of long bone osteomyelitis. Bone Joint Res.

[bib1.bib7] Hotchen AJ, Wismayer MG, Robertson-Waters E, McDonnell SM, Kendrick B, Taylor A, Alvand A, McNally M (2021). The Joint-Specific BACH classification: A predictor of outcome in prosthetic joint infection. eClinicalMedicine.

[bib1.bib8] Kristensen NK, Manning L, Lange J, Davis JS (2024). External Validation of the Joint-Specific Bone Involvement, Antimicrobial Options, Coverage of the Soft Tissues, and Host Status (JS-BACH) Classification for Predicting Outcome in Periprosthetic Joint Infections: A Cohort of 653 Patients. J Arthroplasty.

[bib1.bib9] Kurtz S, Mowat F, Ong K, Chan N, Lau E, Halpern M (2005). Prevalence of primary and revision total hip and knee arthroplasty in the United States from 1990 through 2002. J Bone Joint Surg Am.

[bib1.bib10] McNally M, Sousa R, Wouthuyzen-Bakker M, Chen AF, Soriano A, Vogely HC, Clauss M, Higuera CA, Trebše R (2021). The EBJIS definition of periprosthetic joint infection. Bone Joint J.

[bib1.bib11] McPherson EJ, Woodson C, Holtom P, Roidis N, Shufelt C, Patzakis M (2002). Periprosthetic total hip infection: outcomes using a staging system. Clin Orthop Relat Res.

[bib1.bib12] Singh JA (2011). Epidemiology of knee and hip arthroplasty: a systematic review. Open Orthop J.

[bib1.bib13] Tan TL, Goswami K, Fillingham YA, Shohat N, Rondon AJ, Parvizi J (2018). Defining Treatment Success After 2-Stage Exchange Arthroplasty for Periprosthetic Joint Infection. J Arthroplasty.

[bib1.bib14] Tornero E, Morata L, Martínez-Pastor JC, Bori G, Climent C, García-Velez DM, García-Ramiro S, Bosch J, Mensa J, Soriano A (2015). KLIC-score for predicting early failure in prosthetic joint infections treated with debridement, implant retention and antibiotics. Clin Microbiol Infect.

[bib1.bib15] Tsukayama DT, Estrada R, Gustilo RB (1996). Infection after Total Hip Arthroplasty. A Study of the Treatment of One Hundred and Six Infections. J Bone Joint Surg Am.

[bib1.bib16] Wouthuyzen-Bakker M, Sebillotte M, Lomas J, Taylor A, Palomares EB, Murillo O, Parvizi J, Shohat N, Reinoso JC, Sánchez RE, Fernandez-Sampedro M, Senneville E, Huotari K, Barbero JM, Garcia-Cañete J, Lora-Tamayo J, Ferrari MC, Vaznaisiene D, Yusuf E, Aboltins C, Trebse R, Salles MJ, Benito N, Vila A, Toro MDD, Kramer TS, Petersdorf S, Diaz-Brito V, Tufan ZK, Sanchez M, Arvieux C, Soriano A (2019). Clinical outcome and risk factors for failure in late acute prosthetic joint infections treated with debridement and implant retention. J Infect.

